# “Support the strong” or “Help the weak?”: The effects of social comparison and social distance on cooperative behavior in the dictator game

**DOI:** 10.1002/pchj.802

**Published:** 2024-09-28

**Authors:** Qian Sun, Qinglei Li, Jiamin Qian, Shasha Luo, Yongfang Liu

**Affiliations:** ^1^ Department of Psychology Suzhou University of Science and Technology Suzhou Jiangsu China; ^2^ College of Education Science Hubei Normal University Huangshi Hubei China; ^3^ Shanghai Key Laboratory of Mental Health and Psychological Crisis Intervention, School of Psychology and Cognitive Science East China Normal University Shanghai China

**Keywords:** cooperative behavior, evaluation of others' competence, social comparison, social distance, the dictator game

## Abstract

Within an object‐interdependent context, we conducted three experiments to investigate the influence of social comparisons on cooperative behavior, as well as to assess the mediating and moderating effects of related variables. In Experiment 1 (*n* = 207), we examined whether social comparisons impact cooperative behavior toward a comparator in a dictator game task. Here, we specifically focused on the mediating effects of evaluation of others' competence, along with three other potential mediators: self‐competence evaluation, positive emotions, and negative emotions. Following the insights gained from Experiment 1, we proceeded to Experiments 2 (*n* = 279) and 3 (*n* = 298) to further explore whether social distance moderates the mediating effect of evaluation of others' competence. The results of all three experiments consistently indicated that upward (vs. non‐) comparison facilitated cooperative behavior, whereas downward (vs. non‐) comparison hindered it. Furthermore, our findings revealed that evaluation of others' competence served as a mediator between social comparison and cooperative behavior when the comparator (i.e., the cooperative partner) was perceived as being at a far‐distance, whereas the mediating effect of evaluation of others' competence disappeared when social distance was close. These results reveal the pivotal role of evaluating others' competence and social distance in social interactions from the perspective of social comparison, which provides insights into how to promote cooperative behavior.

## INTRODUCTION

“When you see a person of virtue and capability, you should think of emulating and equaling the person; when you see a person of low caliber, you should reflect on your own weak points (见贤思齐焉，见不贤而内自省也).” Traditional Chinese wisdom suggests that social comparison is not just a passive observation but actively guides subsequent decision‐making processes. In an economic game environment, are people inclined to cooperate with the stronger (i.e., the superior) or with the weaker (i.e., the inferior)? Recent studies have attempted to answer this scientific question by focusing on the relationship between social comparison and cooperative behavior (Gong & Sanfey, 2017; Yip & Kelly, 2013), but the findings remain inconclusive. To further make sense of this topic, we examined the effect of social comparison on cooperation within an object‐interdependent context, as well as the mechanism and the boundary of this effect.

### Social comparison and cooperation

People compare themselves not only to others they perceive as being superior to them (i.e., upward social comparison; Collins, 1996) but also to those they perceive as being inferior (i.e., downward social comparison; Buunk et al., 1990). These comparisons encompass both material rewards and spiritual traits, such as competence and personality. Social comparison not only shapes individuals' self‐evaluation but also profoundly influences their interpersonal interaction processes. For instance, research on friendship found that people were more willing to choose the superior (compared to inferior) as friends (Garcia & Tor, 2018).

Moreover, different directions of social comparison can lead to varying levels of cooperation. Existing literature on this topic includes two types of studies. The first category focuses on an object‐dependent context, in which the object of social comparison does not participate in subsequent cooperation. For instance, Yip and Kelly (2013) found feedback on relative performance (i.e., creativity and sincerity) rankings led to a decrease in self‐reported prosocial behavior, regardless of whether the feedback showed better or worse performance than others. Conversely, Zheng et al. (2015) observed that downward (vs. upward/non‐) comparison encouraged willingness to help and donate, no matter the comparisons were based on actual academic performance (Study 1) or a falsified intelligence test (Study 2). Similarly, Ding (2018) manipulated social comparison by recalling relative academic performance in relation to others and found that upward (vs. non‐) comparison made individuals less inclined to cooperate, while downward comparison did not affect cooperation.

The second category further examines the effect of social comparison on cooperative behavior within an object‐interdependent context, in which the object of social comparison also participates in subsequent cooperations. For instance, Gong and Sanfey (2017) found that downward (vs. upward/non‐) comparison based on performance of reaction time led to more cooperative behavior during the subsequent public goods game. Conversely, Nie and Shi (2020) discovered that upward (vs. downward) comparison based on academic performance increased cooperative behavior in both the dictator and public goods games.

Taken together, the above studies suggest that social comparison has a significant impact on individuals' cooperative behavior, although the specific effect remains inconclusive. Furthermore, object‐interdependent contexts may relatively better capture the complex interpersonal dynamics, given that individuals frequently engage in repeated interactions with those that they compare themselves to in real life. Therefore, we focus on this context to gain a deeper understanding of the interplay between social comparison and cooperation.

### Evaluation of others' competence as a mediator

Social comparison serves as a fundamental mechanism for self‐evaluation, self‐enhancement, and self‐improvement (for a recent review, Unkelbach et al., 2023). This process entails comparing oneself to others, leading to cognitive shifts and assessments that shape one's behavior (Corcoran et al., 2011).

However, the underlying mechanism of how social comparison affects cooperative behavior within object‐interdependent contexts is still elusive. In such contexts, decisions to cooperate with either a stronger or a weaker partner are closely tied to how people perceive and evaluate the comparators after the comparison (Gong & Sanfey, 2017). When specific information about a cooperation partner is lacking, individuals tend to rely on subtle cues or hints to infer others' potential for reciprocity (Tan et al., 2016). Given that social comparison was typically manipulated based on relative performance, we believe that people may likely utilize social comparison information to assess others' competence; such assessments, in turn, influence their cooperative behavior toward the comparators. After all, people generally try to maximize their social interactions for their own benefit (Ma et al., 2019), and the competence of one's social partner determines the potential for mutual benefits in any given interaction (Tan et al., 2016).

Some scattered evidence supports our hypothesis. For example, Tan et al. (2017) found that, in the context of the fame and fortune game, individuals tend to cooperate more to earn a good reputation in front of highly competent others. DeScioli and Krishna (2013) discovered that when people perceive one party as being superior to them, they cooperate more in games for the other party's benefit. In modified dictator games, Hackel and Zaki (2018) found that people reciprocated more toward higher‐wealth givers compared with lower‐wealth givers, even when those givers were equally generous. Buchs and Butera (2009) found that college students while choosing with whom to work depended on the complementarity of their strengths. In other words, they preferred those who were stronger in areas where they were weak over those who were stronger in areas where they were strong themselves.

Based on the above findings, we hypothesize the following:

Hypothesis 1 Individuals are more inclined to cooperate with an upward (vs. non‐) comparator and less inclined to cooperate with a downward (vs. non‐) comparator.

Hypothesis 2 How individuals evaluate the competence of others with whom they interact mediates the influence of social comparison on cooperative behavior.

### Social distance as a moderator

Social interactions inherently involve both self and others. When deliberating on related topics such as social comparison and cooperation, it is essential to take into account social distance—the closeness or remoteness of social relationships between oneself and others (Law et al., 2022; Unkelbach et al., 2023).

Numerous studies consistently indicated that social distance plays a significant role in determining an individuals' willingness to cooperate with others. Specifically, individuals tend to prefer cooperating with those who are socially close compared to those who are socially distant (Osinski et al., 2021; Su et al., 2018). This preference can be attributed to the differing likelihoods of obtaining long‐term benefits and maintaining long‐term interactive relationships when dealing with individuals at different social distances (Law et al., 2022). For instance, cooperation with socially close individuals is typically characterized by higher levels of trust (Fiedler et al., 2011) and lower sense of being exploited (Guéguen & Fischer‐Lokou, 2004), making cooperation with close others more appealing. Conversely, the perceived uncertainty and potential risks associated with cooperating with socially distant individuals may explain the reluctance to engage in such interactions.

The social comparison–cooperation link may differ in different social distance conditions. From an evolutionary perspective, early human beings had to help each other within the group to survive (Hamilton, 1964; Nowak, 2006). Therefore, cooperation among individuals who are closer in the social distance is universal and less likely to be affected by social comparison. Moreover, the mediating mechanism (i.e., social comparison → evaluation of others' competence → cooperative behavior) may vary depending on social distance conditions. Firstly, social distance can modulate the initial stages of this process. Prior research indicates that individuals tend to exhibit greater affective sharing and self‐involvement when interacting with close others (Muller‐Pinzler et al., 2016). This closeness may lead to a cognitive bias in assessing others' strengths and weaknesses, as individuals may disproportionately disregard the true value of these attributes. Furthermore, Liu et al. (2021) found that close social distances tend to elicit a stronger neural response, specifically an increased amplitude of FRN (feedback‐related negativity), which is indicative of a heightened evaluation of others' abilities. These findings suggest that social distance may directly impact how significantly one perceives the competence of others. Secondly, social distance can also influence the subsequent stages of the cognitive mechanism. According to Tan et al. (2016), in contexts involving reputation and profit, others' strengths and weaknesses are related to the objective possibility of the benefits they may provide, whereas social distance is related to the subjective possibility that others will provide benefits. Both these possibilities—objective and subjective—shape individuals' cooperative behavior. When they coexist, complex interaction effects may emerge. Tan et al. (2017) further explored this interaction and found that cooperation (i.e., preference of reputation) in reputation‐profit game was influenced by the interactive effect of social distance and partner's ability. More specifically, participants cooperated more with others when the social distance between them and their interaction partners is closer, as opposed to when it is farther. This effect, however, was only observed when the partner's ability is low.

Based on the aforementioned path analyses, we hypothesize the following:

Hypothesis 3 Evaluation of others' competence mediates the influence of social comparison on cooperative behavior under the condition of far‐distance, whereas the mediating effect of evaluation of others' competence disappears or is attenuated under the condition of close‐distance.

### Overview of the present research

**FIGURE 1:**
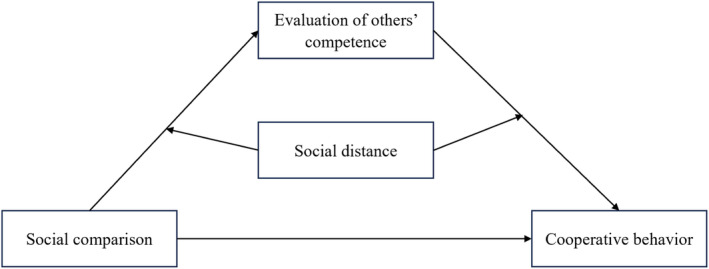
Conceptual moderated mediation model.

We conducted three experiments to test the hypothetical moderated mediation model (Figure 1). In Experiment 1, we tested whether evaluation of others' competence mediates the relationship between social comparison and subsequent cooperative behavior in dictator game tasks. Building on the insights gained from Experiments 1, 2, and 3 further explored whether social distance would moderate the underlying cognitive processes. Across the three experiments, we used the number of tokens that participants allocated to the comparator in a dictator game to measure their cooperative behavior. Additionally, to enhance the diversity and generalizability of the research, in Experiments 2 and 3, we employed distinct manipulation methods of social comparison, as well as measurements of evaluation of others' competence.

## EXPERIMENT 1

### Method

#### Participants and design

A prior power analysis suggested that at least 207 participants are required to observe a significant main effect in a one‐way analysis of variance (ANOVA), assuming a medium effect size (*f* = 0.25) with sufficient power (1–*β* > .90). We recruited 207 college students (118 female; *M*_age_ = 23.16, *SD* = 2.83) through the official recruitment platform of East China Normal University and randomly assigned them to one of the three conditions (Social comparison: upward comparison/non‐comparison/downward comparison). Each participant received five Chinese yuan (ca. 0.70 USD) for taking part in the experiment.

#### Materials and procedures

Participants were invited to take part in a lab experiment on social interactions. They were informed that this experiment contained test questions with instructions, and that data from those who failed the test would be invalid. Participants firstly completed social comparison manipulation and subsequently completed measurements in the following order: self‐competence evaluation, evaluation of others' competence, positive emotions, negative emotions, and cooperative behavior. The specific materials and procedures are described below.

1. Social comparison manipulation. We manipulated social comparison by presenting participants with varying descriptions of academic performance (see Appendix A), which was adapted from Stapel and Koomen (2005). Participants were randomly assigned to different social comparison conditions and required to read text passages detailing the academic situation of their classmate, “Zhan.” In the upward comparison condition (*n* = 69), Zhan was portrayed as someone who finds studying easy, is serious about learning, gets excellent grades, has a wide range of interests, actively participates in activities, and has won various awards. Conversely, in the downward comparison condition (*n* = 67), Zhan was described as someone who has learning difficulties, has a poor attitude about learning, scores ordinary grades, shows little interest in learning new things, does not actively participate in activities, and never wins awards. In the non‐comparison condition (*n* = 70), participants did not receive any such descriptive material. Following this manipulation, participants completed a test titled “I think Zhan is better than me,” using a five‐point Likert scale (1 = *strongly disagree*, 5 = *strongly agree*) for scoring.
2. Evaluation of others' competence. We measured how participants evaluate their comparator's competence using a five‐point Likert scale (1 = *strongly disagree*, 5 = *strongly agree*). The specific item used was that “I believe Zhan's competence is very strong.”
3. Alternative mediators' measurement: *Self‐competence evaluation*. Social comparison not only serves as an inherent driving force for individuals to evaluate themselves but also emerges as a consequence of social comparison process (Gerber et al., 2018). Consequently, it influences individuals' assessment of their own competence, which in turn may affect their subsequent willingness to cooperate (Gong & Sanfey, 2017). To control for its effect, we assessed participants' self‐perception of their own competence using a five‐point Likert scale (1 = *strongly disagree*, 5 = *strongly agree*). The specific item used was that “I believe my competence is very strong.”
*Positive and negative emotions*. Social comparison frequently triggers emotional reactions (Boecker et al., 2022; van de Ven, 2017). Specifically, upward comparison usually evokes negative emotions such as envy and a sense of threat (Boecker et al., 2022; Dunn et al., 2012; Moran & Schweitzer, 2008; van de Ven, 2017), while downward comparison typically elicits positive emotions like happy‐for‐ness (i.e., an emotion mixed with sympathy, joy, and gratitude) and self‐admiration (Boecker et al., 2022; Smith, 2000; Visconti et al., 2013). Importantly, research indicated that emotions mediate the relationship between social comparison and cooperation in object‐dependent contexts (Miao et al., 2021). This suggests that the emotional reactions triggered by social comparison can shape individuals' cooperative tendencies. While object‐interdependent contexts may present distinct challenges and opportunities, positive or negative emotions might serve as mediators in these settings. To control for the influence of emotional factors, we measured participants' emotional state using the Positive and Negative Affect Scale (PANAS) by Watson et al. (1988), which includes 20 questions: 10 positive emotion items (*α* = .88) and 10 negative emotion items (*α* = .91).
4. Cooperative behavior measurement. We measured cooperative behavior using a dictator game, in which the number of tokens allocated to others serves as an indicator of cooperative behavior (Fenzl & Brudermann, 2021; van Dijk & De Dreu, 2021). Participants completed a money distribution task after reading the following instructions for the dictator game task: “You must complete a series of tasks with your classmate, Zhan. Examine the box on the table, which contains 100 plastic tokens. You can now choose how many tokens to keep for yourself and how many to give to Zhan. You are free to make whatever decision you want. Zhan has no authority to reject or reverse your decision. Please fill out the allocation form with your response. While there is no strict time limit, hopefully you will not spend too long. Following the experiment, we will pay you the corresponding reward based on the number of tokens you earned.” After the participants completed the task, the experimenter confirmed that they were unaware of the study's true purpose during the experiment, then explained it to them and paid them the participation fee.

### Results

#### Manipulation checks

We conducted a one‐way ANOVA on manipulation test scores. The results revealed the main effect of social comparison was significant, *F*(2, 204) = 132.58, *p < *.001, *η*_*p*_^2^ = 0.56. Post hoc tests revealed that the upward comparison group (*M* = 2.96, *SE* = 0.06) rated “the degree others are superior to oneself” significantly higher than did the non‐comparison (*M* = 2.38, *SE* = 0.06; *p < *.001, 95% CI [0.41, 0.74]) and downward comparison (*M* = 1.57, *SE* = 0.07; *p < *.001, 95% CI [1.21, 1.55]) groups. Participants in the non‐comparison group rated “the degree others are superior to oneself” significantly higher than did participants in the upward comparison group (*p < *.001, 95% CI [0.64, 0.98]). These results demonstrate the effective manipulation of social comparisons in this experiment.

#### Effect of social comparison on cooperative behavior

**FIGURE 2:**
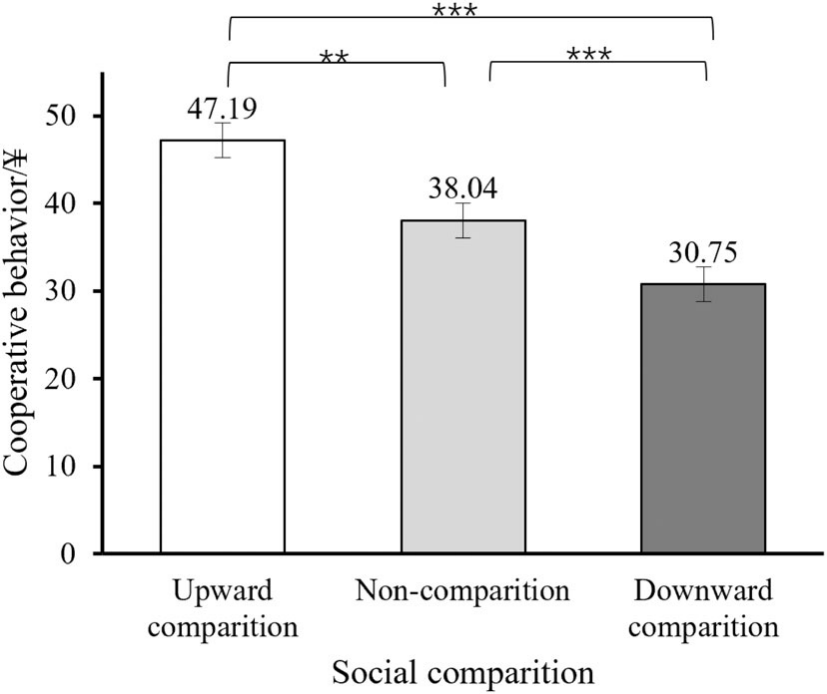
Differences in cooperative behavior under different social comparison conditions. Error bars indicate standard errors; **p < .01; ***p < .001.

We employed a one‐way ANOVA on cooperative behavior (i.e., the number of tokens participants assigned to others). The results (Figure 2) showed that the main effect of social comparison was significant, *F*(2, 204) = 15.13, *p < *.001, *η*_*p*_^2^ = 0.14. Post hoc tests revealed that the upward comparison group (*M* = 46.51, *SE* = 1.81) exhibited significantly higher levels of cooperation compared to both the downward comparison group (*M* = 30.75, *SE* = 2.02; *p < *.001, 95% CI [10.11, 21.43]) and non‐comparison group (*M* = 38.07, *SE* = 1.97; *p = *.003, 95% CI [2.85, 14.04]). Furthermore, the non‐comparison group demonstrated higher levels of cooperation than the downward comparison group (*p = *.01, 95% CI [1.67, 12.98]).

#### Mediating effect test

We used the bootstrapping method for moderation (5000 bootstrap samples, 95% CI, model 4; Hayes, 2018) to test whether the relationship between social comparison (non‐comparison group as the reference condition; indicator coding) and cooperative behavior is mediated by evaluation of others' competence, self‐competence evaluation, positive emotions, and negative emotions.

**FIGURE 3:**
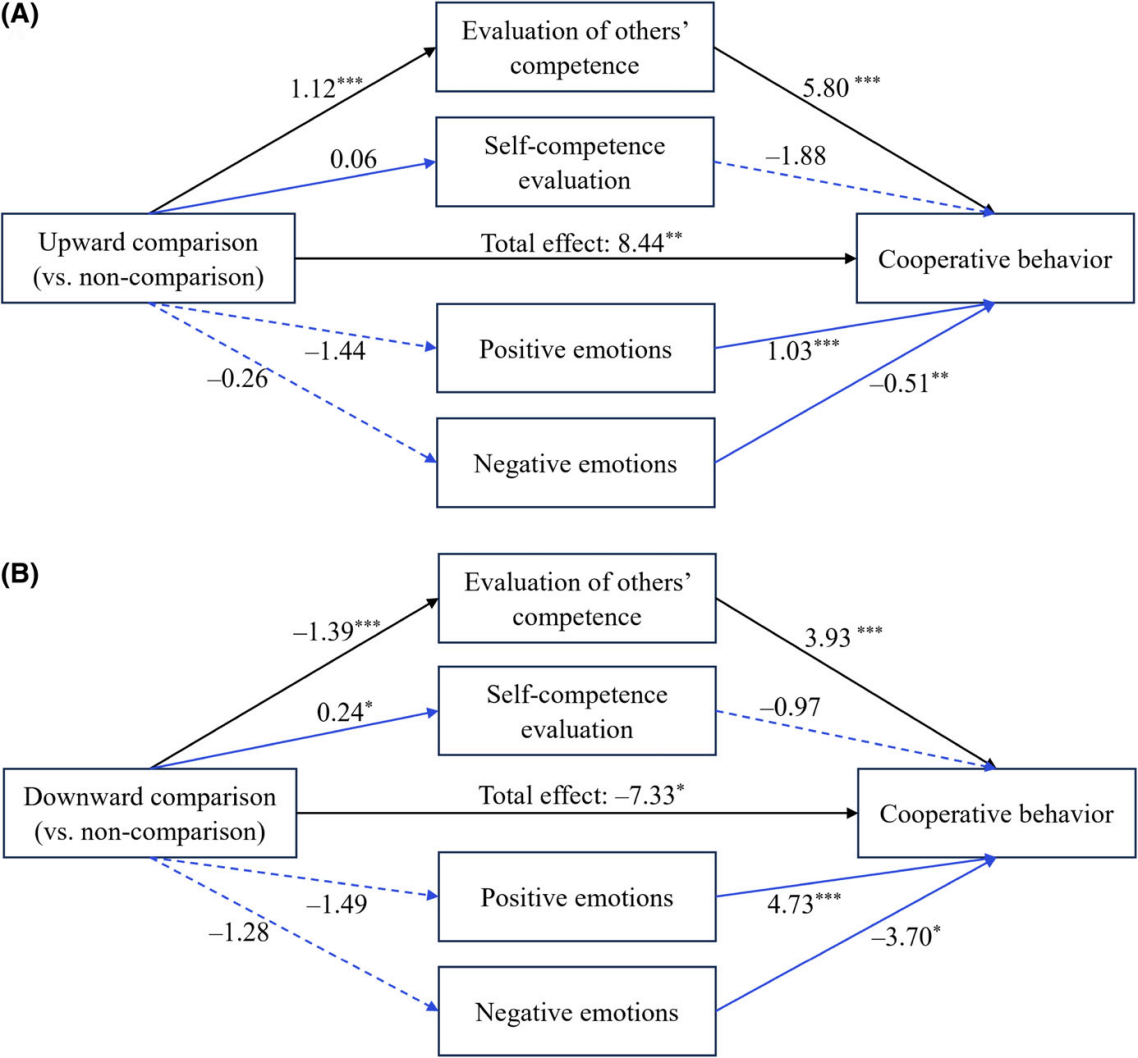
Mediating models in Experiment 1. The coefficients listed in the figure are unstandardized; the blue arrows represent the alternative pathway; *p < .05; ***p < .001.

The results (see Figure 3) showed that (a) evaluation of others' competence significantly mediated both the positive impact of upward (vs. non‐) comparison group on cooperative behavior (*β* = 6.48, 95% CI [2.77, 10.50]) and the negative impact of downward (vs. non‐) comparison group on cooperative behavior (*β* = −8.05, 95% CI [−12.73, −3.84]); (b) self‐competence evaluation did not significantly mediate the impact of either upward (*β* = −.11, 95% CI [−0.78, 0.62]) or downward comparison (*β* = −.46, 95% CI [−1.58, 0.58]) on cooperation, when compared to non‐comparison; (c) positive emotions did not significantly mediate the impact of either upward (*β* = −1.48, 95% CI [−3.50, 0.20]) or downward comparison (*β* = −1.53, 95% CI [−3.65, 0.22]) on cooperation, when compared to non‐comparison; and (d) negative emotions did not significantly mediate the impact of either upward (*β* = .13, 95% CI [−1.22, 1.30]) or downward comparison (*β* = .64, 95% CI [−0.44, 2.11]) on cooperation when compared to non‐comparison.

Moreover, the results showed that neither the direct effect of downward comparison on cooperation (*β* = 2.08, 95% CI [−4.55, 8.70]) nor the direct effect of upward comparison on cooperative behavior was significant (*β* = 3.42, 95% CI [−2.67, 9.51]) when mediating variables were included in the model.

### Discussion

The findings from Experiment 1 indicated that participants in the upward (vs. non‐) comparison group perceived others to be more competent and thus more willing to cooperate with others in the dictator game, while participants in the downward (vs. non‐) comparison group perceived others as being less competent and thus more reluctant to cooperate with others. Furthermore, we did not observe any significant mediating effects from alternative mediators. We return to these observations in the General Discussion.

These results eliminated alternative explanations and supported our hypotheses that evaluation of others' competence serves as a mediator in the relationship between social comparison and cooperation. To test the robustness of our hypothetical mediating mechanism and further examine the moderating role of social distance, we conducted Experiment 2. In this experiment, we primarily concentrated on the mediating role of the evaluation of others' competence, without incorporating measurements of the other three potential mediators.

## EXPERIMENT 2

### Method

#### Participants and design

A prior power analysis determined that a minimum of 206 participants are required to detect a statistically significant interaction effect in a two‐way ANOVA, assuming a medium effect size (*f* = 0.25) with sufficient power (1 − *β* > .90). We ultimately recruited 279 college students (146 female; *M*_age_ = 21.27, *SD* = 2.26) and randomly assigned them to one condition in a 3 (social comparison: upward comparison/non‐comparison/downward comparison) × 2 (social distance: close/far) between‐subject design. There were 46 participants in the upward comparison/close‐distance group, 44 in the non‐comparison/close‐distance group, 46 in the downward/close‐distance group, 48 in the upward comparison/far‐distance group, 47 in the non‐comparison/far‐distance group, and 48 in the downward comparison/far‐distance group. None of the participants had previously taken part in Experiment 1. Each participant received 5 Chinese yuan (ca. 0.70 USD) for taking part in the experiment.

#### Materials and procedures

The specific experimental steps are described below.

1. Social distance manipulation. Participants were assigned to different social distance conditions at random. Participants in the close‐distance group were told the following: “Recall in your mind a classmate with whom you have a good relationship, write down his/her last name, and describe your relationship in one sentence (e.g., he/she is my roommate).” Participants in the far‐distance group were told the following: “You have a classmate named Zhan who you do not know, and he will complete a task with you.” Then, they were asked to use the Inclusion of Other in the Self (IOS) scale to select the image that best described their relationship with their classmate (Aron et al., 1992; Appendix B).
2. Social comparison manipulation. The manipulation technique was the same as that in Experiment 1. However, the difference was that in the close‐distance group, participants were provided the following instructions: “Please read the following description carefully and try to imagine that it is about your above‐mentioned classmate.” In the far‐distance group, participants were told the following: “Please read the following description carefully and try to imagine that this description is about the student described above, Zhan.”
3. The evaluation of others' competence was measured in the same way as in Experiment 1.
4. The measurement of cooperative behavior was the same as that in Experiment 1.

### Results

#### Manipulation checks

##### Manipulation checks of social comparison

A one‐way ANOVA on manipulation test scores indicated that the main effect of social comparison was significant, *F*(2, 276) = 40.66, *p < *.001, *η*_*p*_^2^ = 0.23. Post hoc tests revealed that the upward comparison group (*M* = 3.35, *SE* = 0.07) rated “degree of others' superiority to themselves” significantly higher than did the non‐comparison (*M* = 3.06, *SE* = 0.07; *p = *.003, 95% CI [−0.49, −0.10]) and downward comparison (*M* = 2.90, *SE* = 0.07; *p < *.001, 95% CI [0.67, 1.05]) groups. Participants in the non‐comparison group rated “the degree of others' superiority to themselves” significantly higher than did participants in the downside comparison group (*p < *.001, 95% CI [0.37, 0.75]). This demonstrated that the social comparison manipulation was effective in this study.

##### Manipulation checks of social distance

A one‐way ANOVA on IOS scale rating scores showed the main effect of social distance was significant, *F*(1, 277) = 180.18, *p < *.001, *η*_*p*_^2^ = 0.40. Participants were significantly closer to friends (*M* = 4.91, *SE* = 0.11) than strangers (*M* = 2.81, *SE* = 0.11), indicating that the social distance manipulation was effective.

#### Mediation effect test of evaluation of others' competence

We again used the bootstrapping method for moderation (5000 bootstrap samples, 95% CI, model 4; Hayes, 2018) to test whether the relationship between social comparison (non‐comparison group as the reference condition; indicator coding) and cooperative behavior is mediated by evaluation of others' competence. The results (Table 1) showed that evaluation of others' competence significantly mediated the negative effect of upward (vs. non‐) comparison on cooperative behavior (*β* = 1.02, 95% CI [0.20, 2.23]), as well as the negative effect of downward (vs. non‐) comparison on cooperative behavior (*β* = −3.63, 95% CI [−6.53, −1.20]).

**TABLE 1:** Mediating effect of social comparison on cooperative behavior in Experiment 2.

Variables	Equation (1)			Equation (2)		
	Dependent variable: Evaluation of others' competence (*M*)			Dependent variable: Cooperative behavior (*Y*)		
	*β*	*SE*	*t*	*β*	*SE*	*t*
Social comparison (*X*_1_)	.32*	0.13	2.42	3.69	2.23	1.66
Social comparison (*X*_2_)	−1.15***	0.13	−8.56	2.89	2.48	1.17
Evaluation of others' competence (*M*)				3.14**	0.99	3.19
	*R* ^2^ = .33			*R* ^2^ = .06		
	*F*(2, 276) = 67.52, *p* < .001			*F*(3, 275) = 5.82, *p = *.001		

*Note*: *X*_1_: non‐comparison = 0, upward comparison = 1, downward comparison = 0; *X*_2_: non‐comparation = 0, upward comparison = 0, downward comparison = 1.

* *p < *.05;

** *p < *.01;

*** *p < *.001.

#### Moderated mediation effect test

**FIGURE 4:**
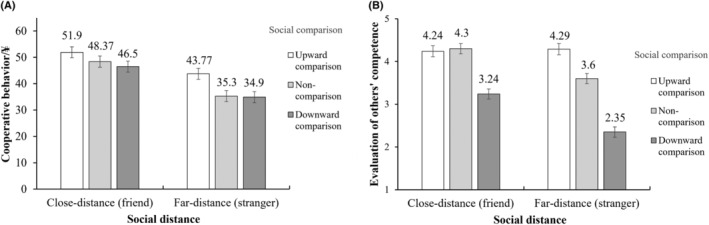
The effects of social comparison and social distance on cooperative behavior (A) and evaluation of others' competence (B) in Experiment 2.

We used the bootstrapping method for moderation (5000 bootstrap samples, 95% CI, model 58; Hayes, 2018) to test whether the mediating effect of evaluation of others' competence on the relationship between social comparison (non‐comparison group as the reference condition) and cooperative behavior is moderated by social distance (*close‐distance* = 0, *far‐distance* = 1). The results (Table 2, Figure 4) revealed a significant interaction effect between upward (vs. non‐) comparison and social distance on the evaluation of others' competence, *β* = .75, 95% CI [0.25, 1.25]. However, the interaction effect between downward (vs. non‐) comparison and social distance on the evaluation of others' competence was not significant, *β* = −.18, 95% CI [−0.68, 0.31]. Additionally, a significant interaction effect between social distance and evaluations of others' competence on cooperative behavior was observed, *β* = 3.31, 95% CI [0.07, 6.55].

**TABLE 2:** Results of the moderated mediation effect model in Experiment 2.

Variables	Equation (1)			Equation (2)		
	Dependent variable: Evaluation of others' competence (*M*)			Dependent variable: Cooperation level (*Y*)		
	*β*	*SE*	*t*	*β*	*SE*	*t*
Social comparison (*X*_1_)	−.82*	0.40	−2.02	3.55	2.15	1.65
Social comparison (*X*_2_)	−.88*	0.40	−2.18	1.12	2.40	0.46
Social distance (*W*)	−.70***	0.18	−3.88	−20.92**	6.38	−3.28
*X*_1_ × *W*	−.18	0.25	−0.73			
*X*_2_ × *W*	.75**	0.25	2.97			
Evaluation of others' competence (*M*)				−3.50	2.82	−1.24
*M* × *W*				3.31*	1.65	2.01
	*R* ^2^ = .41			*R* ^2^ = .14		
	*F*(5, 273) = 38.64, *p* < .001			*F*(5, 273) = 9.21, *p* < .001		

*Note*: *X*_1_: non‐comparison = 0, upward comparison = 1, downward comparison = 0; *X*_2_: non‐comparation = 0, upward comparison = 0, downward comparison = 1; *W*: close‐distance = 0, far‐distance = 1;

* *p < *.05;

** *p < *.01;

*** *p < *.001.

Furthermore, the results indicated that for close‐distance group, the mediating effect of evaluation of others' competence was not significant in either the relationship between upward comparison and cooperative behavior (*β* = .01, 95% CI [−0.31, 0.31]) or in the relationship between downward comparison and cooperative behavior (*β* = .19, 95% CI [−1.68, 2.19]); and for far‐distance group, the mediating effect of evaluation of others' competence was significant in both the relationship between upward (vs. non‐) comparison and cooperative behavior (*β* = 2.16, 95% CI [0.34, 4.64]) and in the relationship between downward (vs. non‐) comparison and cooperative behavior (*β* = −3.91, 95% CI [−7.58, −0.66]).

### Discussion

The results obtained from Experiment 2 replicated those of Experiment 1, strengthening our hypothetical mediating pathway: “social comparison → evaluation of others' competence → cooperative behavior.” Furthermore, the data from Experiment 2 supported our hypothesis that social distance moderates both the initial and subsequent stages of this mediating process. Importantly, the results showed that upward (vs. non‐) comparison promoted cooperative behavior by increasing evaluation of others' competence, whereas downward (vs. non‐) comparison undermined cooperative behavior by decreasing evaluation of others' competence, but only when the comparator was socially distant from the participants.

To further test the robustness of this moderated mediation model, we manipulated social comparison in Experiment 3 by utilizing a task that differed from the one employed in Experiment 2. Moreover, we employed a more comprehensive measurement to assess evaluation of others' competence.

## EXPERIMENT 3

### Method

#### Participants and design

The sample size determination was the same as that in Experiment 2. We ultimately recruited 298 participants (170 female; *M*_age_ = 29.99, *SD* = 6.18) and randomly assigned them to one condition in a 3 (social comparison: upward comparison/non‐comparison/downward comparison) × 2 (social distance: close/far) between‐subject design. There were 49 people in the downward comparison/far‐distance group, 50 in the non‐comparison/far‐distance group, 50 in the upward comparison/far‐distance group, 50 people in the downward/close‐distance group, 49 in the non‐comparison/close‐distance group, and 50 in the upward comparison/close‐distance group. None of the participants had taken part in Experiments 1 and 2 prior to this experiment. Each participant received 5 Chinese yuan (ca. 0.70 USD) for taking part in the experiment.

#### Materials and procedures

**FIGURE 5:**
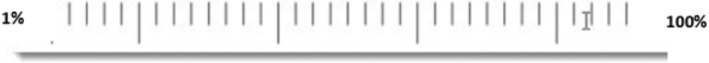
The rectangular ruler of academic ranking.

Except for different methods of social comparison manipulation and measurement of evaluation of others' competence, the other steps and procedures were the same as those in Experiment 2. The specific modifications are as follows:

1. Social comparison manipulation. In this experiment, we manipulated social comparison by recalling relative academic performance, which was adapted from Zheng et al. (2015). Participants were provided with the following instructions: “The rectangular ruler (see Figure 5) below represents a student's ranking position for the entire grade. The top 1% represent first place, and the lowest 100% represent last place. Imagine that the person at the top 1% (bottom 100%) is the best friend you just remembered, and please think back to your grade point average (GPA) last year. Compare yourself to the top 1% (last 100%) good friend (classmate Zhan), and write about where you are (%).” The manipulation was not performed under non‐comparison conditions.

In this paradigm, because the upward comparison other is the highest grade and the downward comparison other is the lowest, objectively, the upward comparison other is stronger than the participant and the downward comparison other is weaker than the participant. Therefore, as in previous study (Zheng et al., 2015), this experiment did not conduct manipulation tests.

2. Evaluation of others' competence. Referring to the measurement of competence in Fiske (2015), participants were asked to use a seven‐point scale (1 = *strongly disagree*, 7 = *strongly agree*) to rate the four dimensions of “competitive,” “capable,” “intelligent,” and “efficient” for others, and the average score was an estimate of others' competence.

### Results

#### Manipulation checks

A one‐way ANOVA on IOS scale rating scores showed that the main effect of social distance was significant, *F*(1, 297) = 658.98, *p < *.001, *η*_*p*_^2^ = 0.69. Participants were significantly closer to friends (*M* = 5.78, *SE* = 0.10) than strangers (*M* = 2.15, *SD* = 0.10), indicating that the social distance manipulation was effective.

#### Mediation effect test

The results (Table 3) showed that evaluation of others' competence significantly mediated the negative effect of upward (vs. non‐) comparison on cooperative behavior (*β* = −7.30, 95% CI [−10.97, −4.12]), as well as the negative effect of downward (vs. non‐) comparison on cooperative behavior (*β* = 3.39, 95% CI [1.84, 5.23]). The results were consistent with those of Experiments 1 and 2.

**TABLE 3:** Results of the mediating effect model of social comparison on cooperative behavior in Experiment 3.

Variables	Equation (1)			Equation (2)		
	Dependent variable: Evaluation of others' competence (*M*)			Dependent variable: Cooperation level (*Y*)		
	*β*	*SE*	*t*	*β*	*SE*	*t*
Social comparison (*X*_1_)	−1.88***	0.16	−11.47	4.85	2.53	1.92
Social comparision (*X*_2_)	.88***	0.16	5.34	−.55	2.20	−0.25
Evaluation of others' competence (*M*)				3.88***	0.75	5.20
	*R* ^2^ = .50			*R* ^2^ = .10		
	*F*(2, 295) = 147.87, *p* < .001			*F*(3, 294) = 11.13, *p* < .001		

*Note*: *X*_1_: non‐comparation = 0, upward comparison = 1, downward comparison = 0; *X*_2_: non‐comparation = 0, upward comparison = 0, downward comparison = 1.

*** *p < *.001.

#### Moderated mediation effect test

**FIGURE 6:**
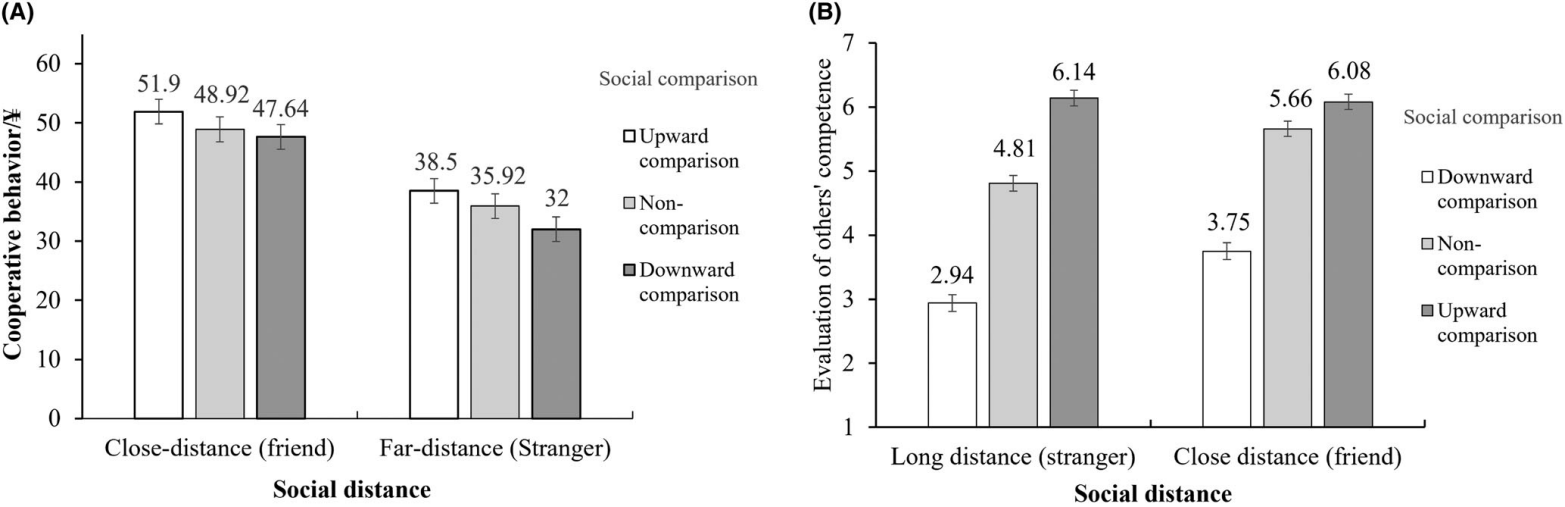
The effects of social comparison and social distance on cooperative behavior (A) and evaluation of others' competence (B) in Experiment 3.

The results (Table 4, Figure 6) showed a significant interaction effect between upward (vs. non‐) comparison and social distance on the evaluation of others' competence, *β* = .92, 95% CI [0.30, 1.54]. However, the interaction effect between downward (vs. non‐) comparison and social distance on the evaluation of others' competence was not significant, *β* = .05, 95% CI [−0.57, 0.67]. Additionally, a significant interaction effect between social distance and evaluations of others' competence on cooperative behavior was observed, *β* = 2.12, 95% CI [0.20, 4.04].

**TABLE 4:** Results of the moderated mediation effect model in Experiment 3.

Variables	Equation (1)			Equation (2)		
	Dependent variable: Evaluation of others' competence (*M*)			Dependent variable: Cooperation level (*Y*)		
	*β*	*SE*	*t*	*β*	*SE*	*t*
Social comparison (*X*_1_)	−.51	0.50	−1.02	0.11	2.00	0.05
Social comparison (*X*_2_)	−1.97***	0.50	−3.95	2.10	2.31	0.91
Social distance (*W*)	−.86***	0.22	−3.85	−23.06**	5.07	−4.55
*X*_1_ × *W*	.05	0.32	0.16			
*X*_2_ × *W*	.92**	0.31	2.92			
Evaluation of others' competence (*M*)				−0.67	1.66	−0.41
*M* × *W*				2.12*	0.98	2.17
	*R* ^2^ = .54			*R* ^2^ = .27		
	*F*(5, 292) = 69.73, *p < *.001			*F*(5, 292) = 21.62, *p < *.001		

*Note*: *X*_1_: non‐comparison = 0, upward comparison = 1, downward comparison = 0; *X*_2_: non‐comparision = 0, upward comparison = 0, downward comparison = 1; *W*: close‐distance = 0, far‐distance = 1.

* *p < *.05;

** *p < *.01;

*** *p < *.001.

Furthermore, the results indicated that for close‐distance, the mediating effect of evaluation of others' competence was not significant either in the relationship between upward comparison and cooperative behavior (*β* = .60, 95% CI [−0.02, 1.48]) or in the relationship between downward comparison and cooperative behavior (*β* = −2.77, 95% CI [−6.25, 0.01]). For far‐distance, the mediating effect of evaluation of others' competence was significant in both the relationship between upward comparison and cooperative behavior (*β* = 4.74, 95% CI [2.15, 7.43]) and in the relationship between downward comparison and cooperative behavior (*β* = −6.65, 95% CI [−10.57, −3.10]).

### Discussion

In Experiment 3, using distinct experimental tasks, both the mediating effect of evaluation of others' competence and the moderating effect of social distance exhibited patterns consistent with those observed in Experiment 2. This consistency backs our hypotheses and demonstrates the relative reliability of our conceptual moderated mediation model.

## GENERAL DISCUSSION

Within an object‐interdependent context, we explored the mechanism (Experiments 1, 2, and 3) and the boundary (Experiments 2 and 3) in the effect of social comparison on cooperative behavior. Across three experiments, we consistently observed that upward (vs. non‐) comparison facilitated cooperative behavior, whereas downward (vs. non‐) comparison hindered it. Furthermore, as hypothesized, evaluation of others' competence served as a mediator in the causal effect of social comparison on cooperative behavior. Importantly, this mediating effect was only evident when the comparator (i.e., the cooperative partner) was perceived as being at a far‐distance.

### Social comparison's effect on cooperative behavior

Do people “support the strong” or “help the weak”? The three experiments in this study reveal that, when people make cooperative decisions in an economic game task that is directly tied to personal benefits and losses, they prefer to support those who are stronger, rather than help those weaker, which supports the findings of Nie and Shi (2020) but contradicts those of Gong and Sanfey (2017). This could be because of the various methods of manipulating social comparisons used in different studies. However, when compared to Nie and Shi (2020), this study added a non‐comparison group as a control group (Gerber et al., 2018; Möller & Marsh, 2013), and included different social comparison measurements (Stapel & Koomen, 2005; Yip & Kelly, 2013), which not only eliminated the distractions that social comparisons themselves may bring but also increased the robustness of the results.

The above results also support the insight of reciprocity. People are willing to pay more for those who are more likely to provide them with higher returns (Ma et al., 2019), and when individuals use social comparison to identify who they perceive as being able to provide them with higher returns, they are more likely to cooperate with those individuals. In a real economic game, the “stronger” has the potential to provide more returns (i.e., greater social exchange value); thus, people are more willing to cooperate with them. While upward social comparison may lead to negative emotional experiences such as decreased self‐evaluation and increased shame (van de Ven, 2017), and downward social comparison may lead to positive emotions such as improved self‐evaluation and increased empathy (Boecker et al., 2022; Smith, 2000), these are insignificant relative to the likelihood of a realistic return.

### The mediating role of evaluation of others' competence

Nie and Shi (2020) did not investigate why people are more willing to cooperate based on upward rather than downward comparison. Experiment 1 of this study found that evaluation of others' competence mediates the influence of social comparison on cooperative behavior, while self‐competence evaluation and positive and negative emotions do not play mediating roles. This finding was further corroborated by Experiments 2 and 3, which confirmed the mediating role of evaluation of others' competence in the relationship between social comparison and cooperative behavior. These results are consistent with previous findings (Chatzisarantis et al., 2016; Diel et al., 2021). Furthermore, our findings explain why people prefer to cooperate with those they perceive as “the strong.” In the context of a real economic game, strength is synonymous with competence, which holds significant value (Ehrke et al., 2021; Fiske, 2018; Hackel & Zaki, 2018). People make cooperative decisions in this context based on the competency‐first principle (DeScioli & Krishna, 2013; Eisenbruch et al., 2016; Ma et al., 2019). Even if evaluations of others' competence are based on academic achievement clues unrelated to the subsequent game task, this information will be used to make inferences regarding the subsequent cooperative task (Palmer & Peterson, 2020). However, this result also supports the above inference that self‐evaluation and changes in positive and negative emotions are insignificant for such tasks.

Interestingly, Smith (1776b/2015) proposed the “principle of empathy” in his early *Theory of Moral Sentiments*, arguing that people would sympathize with and cooperate with the weak. However, in his later work, *The Wealth of Nations* (Smith, 1776a/2015), he famously proposed the rational economic man hypothesis, arguing that people would only cooperate with the strong for self‐interest, a contradiction later dubbed as “Smith's paradox.” This study showed that people value profit more than cooperative consideration (e.g., warmth, compassion, love) within an object‐interdependent context. Although this is unavoidably regrettable, it also represents reality.

### The moderating role of social distance

Experiments 2 and 3 of this study consistently showed that the above results would be applicable under far‐distance conditions and less applicable under close‐distance conditions. Social distance moderates the influence of social comparison on evaluation of others' competences and the influence of evaluation of others' competences on cooperative behavior. When the social distance between an individual and others is large, the above results hold true in terms of the role of social comparison in evaluating the competence of others. When an individual's social distance from others is close, the role of social comparison in evaluation of others' competence is weakened, and the difference in competence evaluation between upward and non‐comparative others disappears.

According to the self‐evaluation maintenance model (Tesser, 1988), people have a fundamental need to maintain a positive self‐evaluation, and psychological intimacy between an individual and comparator leads to two distinct processes: contrast and reflection. The former uses the competence of others as the standard of self‐evaluation, and the closer the relationship, the more the excellent performance of others will threaten an individual's positive self‐evaluation. The latter views the competence of others as a representation or reflection of the self, and the closer the relationship, the better the performance of others will uphold an individual's positive self‐evaluation (Pemberton & Sedikides, 2001; Tesser et al., 1988). According to Tesser et al. (1988), contrast effects occur when an individual is compared to a close other in an important dimension and their high competence level threatens the individual's self‐evaluation. Academic competency was the social comparison factor used in this study, and it can threaten individuals' self‐evaluation. When individuals upwardly compare themselves with others with close social distance, they evaluate the upward comparators as less competent because of the motive of self‐evaluation maintenance, thereby shrinking the gap between the upward comparison and the non‐comparison groups.

Regarding the role of evaluation of others' competence on cooperative behavior, both Experiments 2 and 3 show that individuals are more likely to be with highly competent others compared with those who are less competent when social distance is far. However, when social distance is close, differences in cooperative behavior based on others' competence disappear. This result is consistent with past findings (Tan et al., 2017).

According to the construal‐level theory of psychological distance (Trope & Liberman, 2010), people use increasingly higher levels of construal to represent an object as the psychological distance from the object increases. When social distance is far, people interpret others at a higher level to extract their core features and form abstract mental construal (Liberman & Trope, 2014; Trope & Liberman, 2010). In this case, the core characteristics (competence) of others in cooperative tasks become the only clues, which in turn affect the level of cooperation between individuals and others. Conversely, at close social distance, people interpret others at a lower level to extract their more diverse features and establish mental construal (Trope & Liberman, 2010). In this case, others' competence is not the only clue that can be applied when making cooperative decisions, that is, the relationship between individuals and others may also play a role.

In summary, according to this study's findings, in the context of economic games with stakes, people choose the most competent others based on realistic considerations of the exchange of interests; however, less social distance helps to weaken this tendency. In theory, these findings add to and promote existing relevant research, while also clarifying the potential mechanisms and boundary conditions of upward and downward social comparisons influencing cooperative behavior in economic games. In practice, the findings of this study have implications for how people can improve the cooperative behavior of others and themselves. Specifically, improving one's own competence or strengths is likely to be the most effective method, while closing the social distance between oneself and others is another.

### Limitations and future research directions

Some limitations and future directions should be noted. Firstly, we did not include the measurement of potential benefits inferred from others' competence, even though we hypothesize that this may account for why evaluation of others' competence mediates social comparison and cooperative behavior. Further research could investigate this pathway by explicitly considering the sequence of “social comparison → evaluations of others' competence → reward based on competence evaluations → cooperative behavior” to provide additional empirical evidence. Secondly, the cooperative game paradigm used in this study was the dictator game, and the task of the two‐person economic game was a unilateral decision made by the participant as a dictator; the other party did not have decision‐making power. This paradigm was chosen to eliminate the influence of other people's reactions (i.e., partners' willingness to cooperate or reactions to unfair results) on individual cooperative behavior and instead examine cooperative decisions based solely on an individual's own reactions after experiencing social comparison. Thus, it remains unknown whether the conclusions reached in this study can be generalized to other cooperative game scenarios. For example, in ultimatum games and public goods games, both parties must participate in cooperative tasks (Gong & Sanfey, 2017; Nie & Shi, 2020). Future research can combine various cooperative economic game tasks with social comparison paradigms, consider the influence of others' reactions, and identify the internal reasons for the inconsistent conclusions of previous studies about social comparison's influence on cooperative behavior. Thirdly, future research could investigate the moderating roles of other external factors, such as power and other people's personality types. Finally, while the social comparison paradigm in this study used higher academic level indicators related to college students' self‐worth (Stapel & Koomen, 2005; Yip & Kelly, 2013), other indicators, such as social status or appearance, could be added to future studies to manipulate social comparisons and increase the robustness of the experimental results.

## CONCLUSIONS

Cooperative behavior is influenced by social comparison in object‐interdependent contexts. Individuals' cooperation with those they evaluate based on upward comparison, non‐comparison, and downward comparison is significantly reduced in turn. Moreover, evaluation of others' competence mediates the influence of social comparison on cooperative behavior. Finally, social distance moderates the influence of social comparison on evaluation of others' competences and the influence of evaluation of others' competences on cooperative behavior.

## CONFLICT OF INTEREST STATEMENT

The authors declare there are no conflicts of interest.

## ETHICS STATEMENT

The questionnaire and methodology for this study was approved by the Human Research Ethics committee of the East China Normal University (Ethics approval number: HR 431‐2019), and written informed consent was obtained from the participants.

## INFORMED CONSENT

Informed consent was obtained from all individual participants included in the study.

## Data Availability

All data, models, and code generated or used during the study appear in the submitted article.

## APPENDIX A SOCIAL COMPARISON MANIPULATION MATERIALS IN EXPERIMENTS 1 AND 2

### A.1 | Upward comparison

Please first recall your academic level during your university years, and carefully read the following description of another student, Zhan. Compare yourself with Zhan and complete the following questions.

When Zhan attended university, studying was very relaxed. He attends classes on time, never misses class, actively makes his own study plans, and arranges and strictly implements work and rest time. In addition to the tasks assigned by teachers, he also arranges many learning tasks himself. He is very interested in learning, his academic life is very fulfilling, and he likes to discuss problems and exchange ideas with teachers. As long as there are lectures in school, he will listen to them. He also actively participates in various learning competitions held by the school and has won many awards. In addition, in his first semester, he passed the CET‐6 and CET‐3 in computers, with excellent grades in each subject, and won the first‐place scholarship.

### A.2 | Downward comparison

Please first recall your academic level during your university years, and carefully read the following description of another student, Zhan. Compare yourself with Zhan and complete the following questions.

When Zhan attended university, he felt that many courses are difficult and finds it difficult to study. The university has a relatively liberal learning atmosphere, so he has a casual attitude toward studying and occasionally skips class. For the homework assigned by teachers, he often procrastinates and his work on them is mediocre. Although he sometimes studies hard, this never lasts long, so he is ranked low in his class. He has little interest in learning and his grades in various subjects are average. He often makes up for his previous lack of studying a day or two before his exams, and only barely passes them. After three attempts, he passed the fourth level of English. He also has not taken the initiative to participate in various competitions organized by the school and has not won any scholarships.

## APPENDIX B INCLUSION OF OTHER IN THE SELF (IOS) SCALE

### B.1 | Close‐distance group

Below are seven pairs of circles with different degrees of overlap, one representing yourself and the other representing your friend mentioned above. The intersecting part of the circles represents the closeness of your relationship, with a larger intersecting area indicating a closer relationship. 1 represents a very distant relationship between yourself (自己) and the other person (对方), while 7 represents a very close relationship. Please select the graph that best fits your relationship with your friend and write down the corresponding number.

### B.2 | Far‐distance group

Below are seven pairs of circles with different degrees of overlap, one representing yourself and the other representing “Zhan” mentioned above. The intersecting part of the circles represents the closeness of your relationship, with a larger intersecting area indicating a closer relationship. 1 represents a very distant relationship between yourself (自己) and the other person (对方), while 7 represents a very close relationship. Please select the graph that best fits your relationship with Zhan and write down the corresponding number.
